# Evaluation of the prognostic value of tumor-infiltrating lymphocytes in triple-negative breast cancers

**DOI:** 10.18632/oncotarget.10054

**Published:** 2016-06-15

**Authors:** Tian Tian, Miao Ruan, Wentao Yang, Ruohong Shui

**Affiliations:** ^1^ Department of Pathology, Fudan University Shanghai Cancer Center, Shanghai, China; ^2^ Department of Oncology, Shanghai Medical College, Fudan University, Shanghai, China

**Keywords:** tumor-infiltrating lymphocytes, breast cancer, triple-negative, prognostic factor, survival

## Abstract

Tumor-infiltrating lymphocytes (TILs) may be associated with clinical outcome in triple-negative breast cancers (TNBCs). However, lacking of standardized methodologies in TILs evaluation has hindered its application in clinical practice. To evaluate the prognostic role of TILs scored by methods recommended by International TILs Working Group 2014, we performed a retrospective study of TILs in 425 primary invasive TNBCs in a Chinese population with a median follow-up of 4 years. Intratumoral TILs (iTILs) and stromal TILs (sTILs) were scored respectively. The associations between TILs and disease-free survival (DFS), distant disease-free survival (DDFS) and overall survival (OS) were evaluated with COX models. ITILs were not associated with prognosis. Higher sTILs were associated with better prognosis; for every 10% increase in sTILs, a 5% reduction of risk of recurrence or death (*P* < 0.001), 5% reduction of risk of distant recurrence (*P* < 0.001), and 4% reduction of risk of death (*P* = 0.002) were observed. Multivariate analysis confirmed sTILs to be an independent prognostic marker. 3.5% of TNBCs had more than 50% lymphocytes (lymphocyte-predominant breast cancer, LPBC), and associations between LPBC status and prognosis were observed but did not reach statistical significance. TNBCs with more than 20% sTILs had a significantly better prognosis than the patients with no more than 20% sTILs. In conclusion, our study indicated that sTILs scored by methods recommended by International TILs Working Group 2014 were associated with the prognosis of TNBCs. STILs could be an independent prognostic biomarker in TNBCs, increasing sTILs predicting better prognosis.

## INTRODUCTION

Triple-negative breast cancers (TNBCs) have been classified as a breast carcinoma subgroup which is negative for estrogen receptor (ER), progesterone receptor (PgR) and HER2 expression. Clinically, TNBCs present as a group of heterogeneous tumors with various morphology, prognosis, and treatment response. Majority of TNBCs have a higher rate of distant recurrence and a poorer prognosis compared with other breast cancer subtypes [1–3]. Due to the absence of effective targeted therapies, new treatments for TNBCs should be developed currently. Recently, immunologic therapy in breast cancers is upcoming, such as monoclonal antibodies blocking CTLA-4 and PD-1/PD-L1, which may be a new choice in TNBCs treatment in future [4]. Tumor-infiltrating lymphocytes (TILs) in the microenvironment of breast tumors have been proposed to reflect the efficacy to the immune therapy and to predict the prognosis of breast cancers [5–6].

Several studies have demonstrated that high levels of TILs may be associated with a better clinical outcome and may reflect the response to chemotherapy in TNBCs [6–11]. However, methodologies of TILs evaluation in these studies were not standardized, which has hindered its application in clinical practice. To improve the consistency and reproducibility for evaluating TILs in research and clinical practice, the International TILs Working Group issued consensus recommendations of pathologic assessment methods of TILs in 2014 [12]. However, 2015 St. Gallen Consensus didn't accept the application of TILs as a prognostic marker in clinical practice currently [13]. It was suggested that a biomarker could not be recommended for routine use until a standardized approach has been validated in multiple settings [14].

In this study, we carried out a retrospective analysis of TILs in 425 primary invasive TNBCs in a Chinese population. We evaluated stromal tumor-infiltrating lymphocytes (sTILs) as well as intratumoral tumor-infiltrating lymphocytes (iTILs), using the scoring methods recommended by the International TILs Working Group 2014. The aim of our study was to examine the prognostic role of TILs in TNBCs and to evaluate the feasibility of the scoring methods in clinical practice.

## RESULTS

### Baseline clinical characteristics

The clinical characteristics of 425 TNBC patients were listed in Table 1. The mean patients' age was 52 years (interquartile range, 44–59 years). 61.9% of patients have no lymph node metastasis, 17.6% of patients have 1–3 lymph nodes metastasis and 14.8 % of patients have more than 3 lymph nodes involved. 413 (97.2%) TNBCs were diagnosed as invasive carcinoma of no special type and 2.8% were invasive breast carcinoma of special subtypes (metaplastic carcinoma in 4 cases; carcinoma with apocrine differentiation in 6 cases; carcinoma with medullary features in 2 cases). Most patients (80.6%) underwent mastectomy with or without radiotherapy. Breast-conserving surgery was performed in 19.4% of patients and all of them received radiation therapy. 38.8% of patients were treated with anthracyclines-based and 61.2% were treated with anthracyclines + taxanes-based adjuvant chemotherapy.

**Table 1:** Baseline clinical characteristics of sTILs and iTILs in different TNBC groups

Characteristics	N (%)	*P*-value of sTILs	*P*-value of iTILs
Age (years)			
≤ 50	196 (46.1)	0.002^*^	0.17^*^
> 50	229 (53.9)		
Tumor size (cm)			
pT1 (0.1–2.0)	180 (42.4)	0.73^#^	0.19^#^
pT2 (2.1–5.0)	227 (53.4)		
pT3 (>5.0)	12 (2.8)		
Unknown	6 (1.4)		
Nodal status			
pN0 (0)	263 (61.9)	0.08^#^	0.95^#^
pN1 (1–3)	75 (17.6 )		
pN2/N3 (4+)	63 (14.8)		
Unknown	24 (5.7)		
Histological grade			
2	136 (32)	< 0.001^*^	0.005^*^
3	289 (68)		
Histological type			
Invasive carcinoma of	413 (97.2)	0.33^*^	0.25^*^
no special type			
Special subtype	12 (2.8)		
Local treatment			
Mastectomy	247 (59.1)	0.04^#^	0.10^#^
Mastectomy+ RT	86 (21.5)		
BCS + RT	92 (19.4)		
Chemotherapy			
Anthracyclines	165 (38.8)	0.02^*^	0.28^*^
Anthracyclines+Taxanes	260 (61.2)		

Abbreviations: TNBC, triple-negative breast cancer; sTILs, stromal tumor-infiltrating lymphocytes; iTILs, intratumoral tumor-infiltrating lymphocytes; RT, radiotherapy; BCS, breast-conserving surgery.

* Mann-Whitney test;

# Kruskal-Wallis test.

### TILs distribution and association with clinicopathologic features

**Figure 1:**
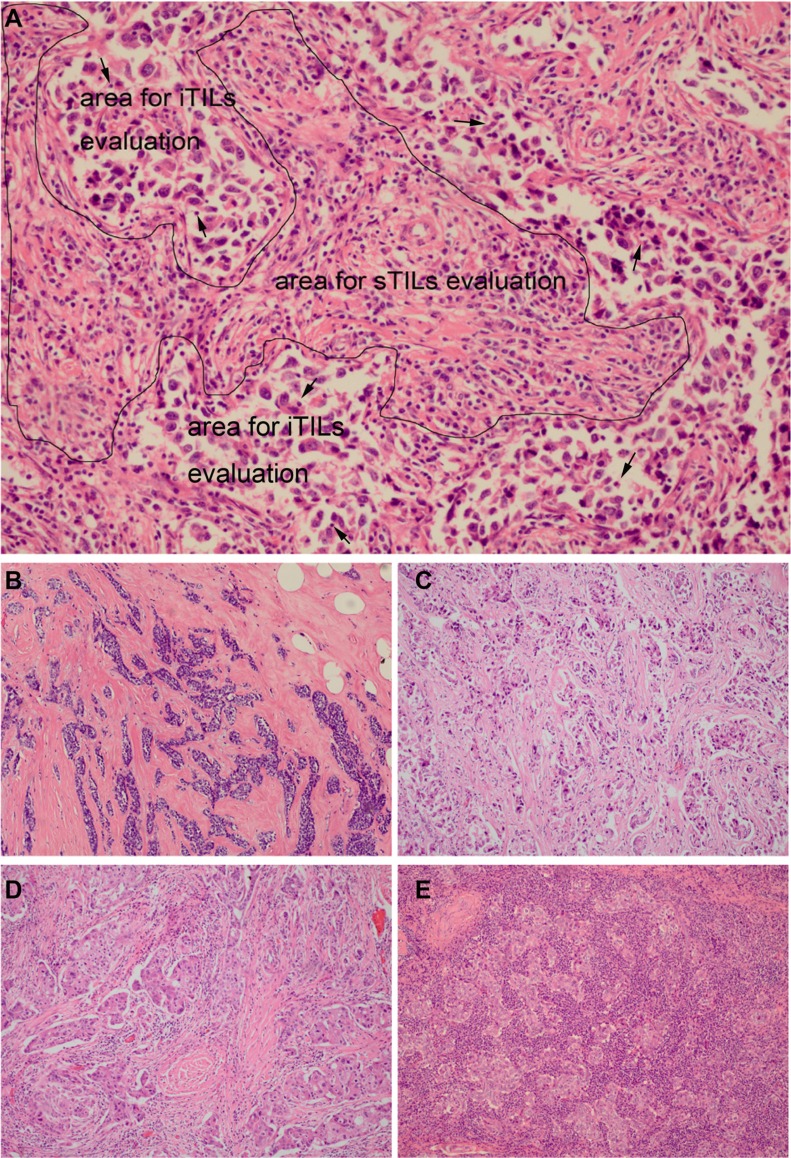
Different scores of tumor-infiltrating lymphocytes (TILs) in TNBCs (**A)** sTILs evaluation area was marked with the black borders and iTILs evaluation area was marked with the black arrow (sTILs 31–40%; iTILs 1–10%); (**B)** sTILs: 0%; (**C)** sTILs: 1–10%; (**D)** sTILs: 11–20%; (**E)** sTILs: 61–70% (A x200 magnification, B, C, D and E x100 magnification).

The distribution of TILs in 425 cases was shown in Table 2. Overall, the average score of sTILs was 14.2% (range: 0–90%); the average score of iTILs was 1.4% (range: 0–30%). Different scores of TILs were shown in Figure 1. STILs were observed in 65.9% of the cases, while iTILs were found in 21.6% of the cases. The correlation between mean scores of sTIL and iTIL was 0.49 (*P* < 0 .001). The distribution of sTILs was skewed, which was concentrated in the range of 0 to 30%. LPBCs (≥ 50% TILs) were seen in only 3.5% of 425 cases. Scoring of sTILs showed an excellent interobserver agreement (ICC 0.95, 95% CI 0.94–0.96, *P* < 0.001), while assessment of iTILs displayed a moderate correlation (ICC 0.46, 95% CI 0.36–0.55, *P* < 0.001).

**Table 2:** Distribution of TILs in TNBCs

Levels	Cancers with sTILs N (%)	Cancers with iTILs N (%)
0–1%	145 (34.1)	333 (78.4)
2–10%	133 (31.2)	89 (20.9)
11–20%	71 (16.7)	1 (0.2)
21–30%	32 (7.5)	2 (0.5)
31–40%	16 (3.7)	0
41–50%	13 (3.3)	0
51–60%	9 (2.0)	0
61–70%	2 (0.5)	0
71–80%	2 (0.5)	0
81–90%	2 (0.5)	0
91–100%	0	0
LPBC status		
LPBC (sTILs ≥ 50%)	15 (3.5)	/
No LPBC (sTILs < 50%)	410 (96.5)	/

Abbreviations: TNBCs, triple-negative breast cancers; TILs, tumor-infiltrating lymphocytes; sTILs, stromal tumor-infiltrating lymphocytes; iTILs, intratumoral tumor-infiltrating lymphocytes; LPBC, lymphocyte-predominant breast cancer.

**Figure 2:**
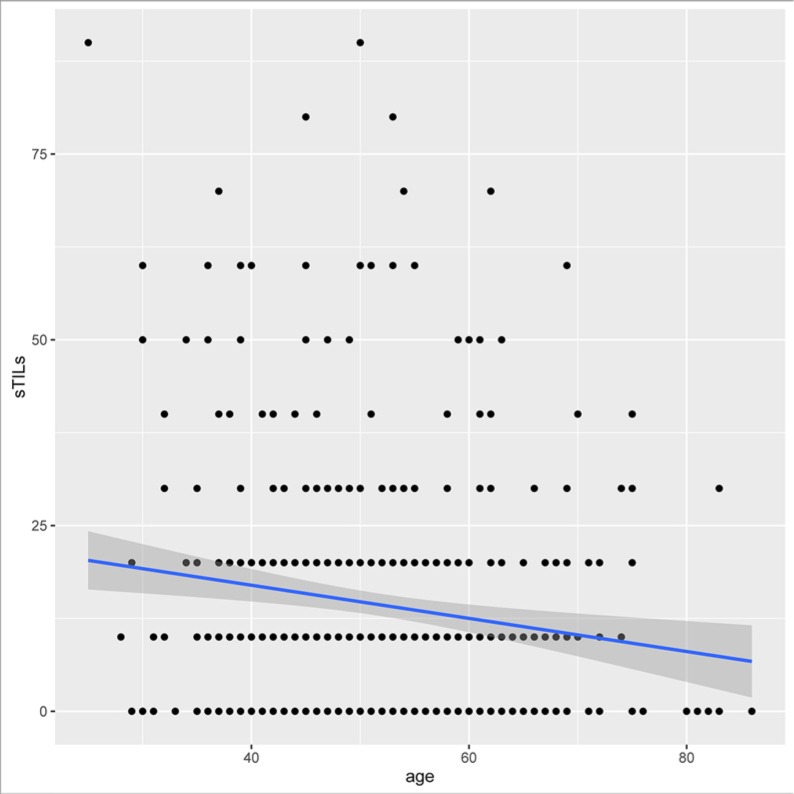
The correlation between sTILs and patients' age in TNBCs Y axis represented the scores of sTILs; X axis represented patient's age (years). STILs scores were negatively associated with patients' age (*r* = −0.14, *P* = 0.003).

Among the clinicopathologic characteristics analyzed, sTILs scores were negatively associated with patients' age (*r* = −0.14, *P* = 0.003, Figure 2, Table 1) and positively associated with higher histological grade (*P* < 0.001, Table 1). STILs scores were higher in TNBCs with younger age or higher histological grade. There was no significant association of sTILs with tumor size (*r* = −0.08, *P* = 0.07) or lymph node metastasis (*r* = −0.007, *P* = 0.88). ITILs were only positively correlated with higher histological grade (*P* = 0.003, Table 1). There were no significant associations of iTILs with age (*r* = 0.002, *P* = 0.97), tumor size (*r* = 0.27, *P* = 0.21) or lymph node metastasis (*r* = 0.08, *P* = 0.21).

### Association of TILs with prognosis

**Figure 3:**
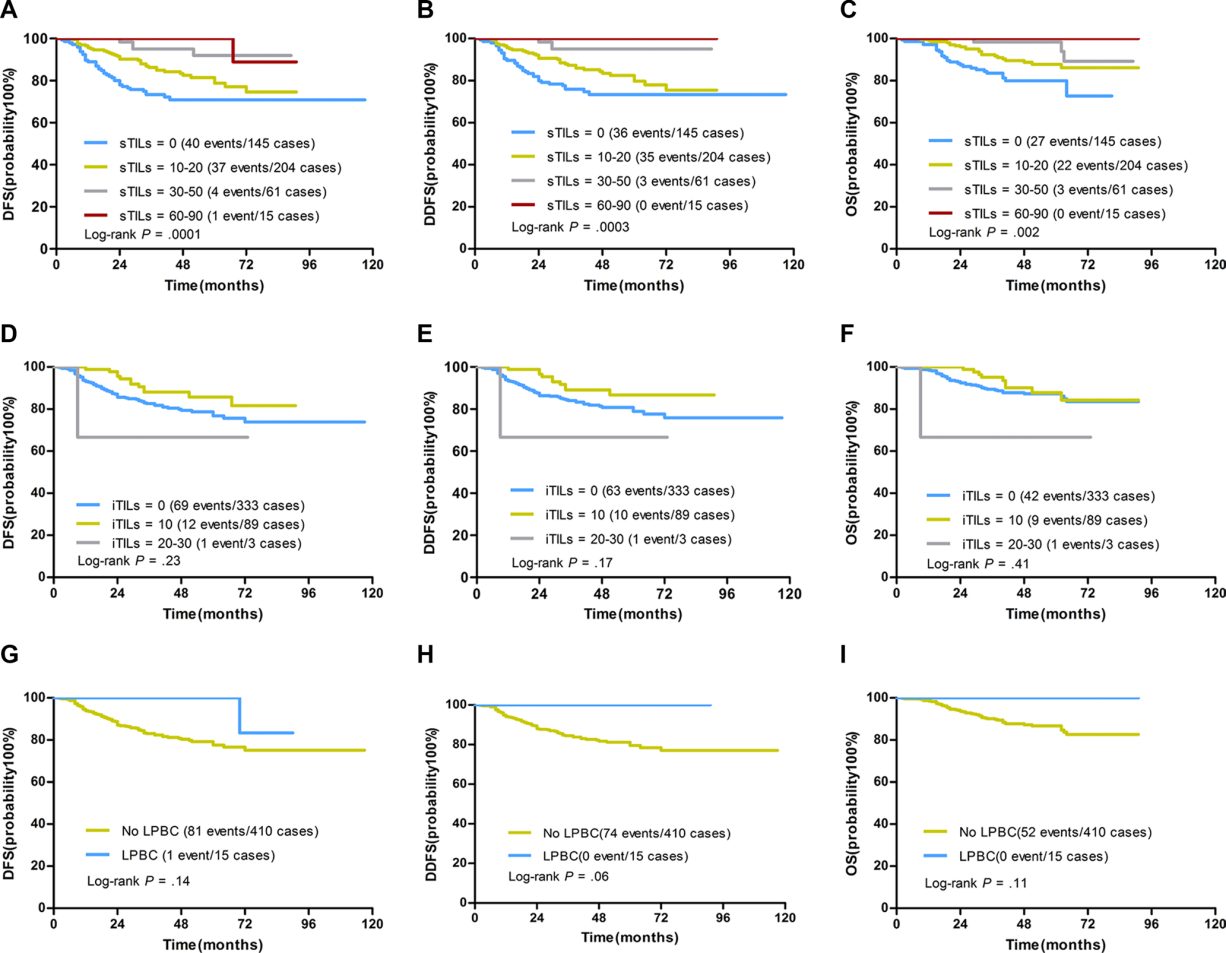
Kaplan-Meier curves for associations of TILs with DFS, DDFS and OS in TNBCs (**A–C)** DFS, DDFS and OS by sTILs (grouped as 0 [defined as 0% to 1%] v 10–20 [1% to 20%] v 30–50 [21% to 50%] v 60–90 [51% to 90%]). STILs scores were significantly associated with DFS, DDFS and OS. (**D–F)** DFS, DDFS and OS by iTILs (grouped as 0 [defined as 0% to 1%] v 10 [1% to 10%] v 20–30 [11%-30%]). ITILs were not associated with DFS, DDFS and OS. (**G-I)** DFS, DDFS and OS by sTILs as a dichotomized variable (LPBC: sTILs ≥ 50%; No LPBC: sTILs < 50%). LPBCs were associated with DFS, DDFS and OS but did not reach significance by Log-rank test (Log-rank *P* values were shown).

The association between TILs and prognosis was analyzed. Among 425 cases, there were 82 DFS events, 74 DDFS events and 52 OS events. There was no significant prognostic association between iTILs and DFS (*P* = 0.53), DDFS (*P* = 0.57) or OS (*P* = 0.61) in univariate analysis (Table 3). Higher sTILs scores were significantly associated with better prognosis. When considered as a continuous variable, sTILs were significantly associated with DFS (HR 0.97; 95% CI 0.95–0.99, *P* = 0.001), DDFS (HR 0.96; 95% CI 0.94–0.98, *P* = 0.001) and OS (HR 0.96; 95% CI 0.94–0.99, *P* = 0.003) in univariate analysis (Table 3). When dichotomized by 50% cutoff, the LPBCs were associated with DFS, DDFS and OS but did not have statistical significance (Table 3). Kaplan-Meier curves of DFS, DDFS and OS visualized the prognostic effect of sTILs, and showed that the LPBC variable did not reach the significance (Figure 3).

**Table 3:** Univariate analysis for associations of TILs with DFS, DDFS and OS in TNBCs

Variables	DFS			DDFS			OS		
	HR	95% CI	*P*-value	HR	95% CI	*P*-value	HR	95% CI	*P*-value
sTILs(per 10% increase)	0.97	0.95–0.99	0.001^*^	0.96	0.94–0.98	0.001^*^	0.96	0.94–0.99	0.003^*^
iTILs(per 10% increase)	0.98	0.91–1.05	0.53	0.98	0.91–1.06	0.57	1.02	0.95–1.10	0.61
LPBC v No LPBC	0.25	0.04–1.83	0.17	0.05	0.01–6.03	0.22	0.05	0.03–13.6	0.29
sTILs ≤ 20% v sTILs > 20%	0.24	0.10–0.60	0.002^*^	0.16	0.05–0.51	0.002^*^	0.23	0.07–0.73	0.01^*^

Abbreviations: TNBCs: triple-negative breast cancers; sTILs, stromal tumor-infiltrating lymphocytes; iTILs, intratumoral tumor-infiltrating lymphocytes; LPBC, lymphocyte-predominant breast cancer; DFS, disease-free survival; DDFS, distant disease-free survival; OS, overall survival; HR, hazard ratio; CI, confidence interval.

* The *P* value is significant.

**Figure 4:**
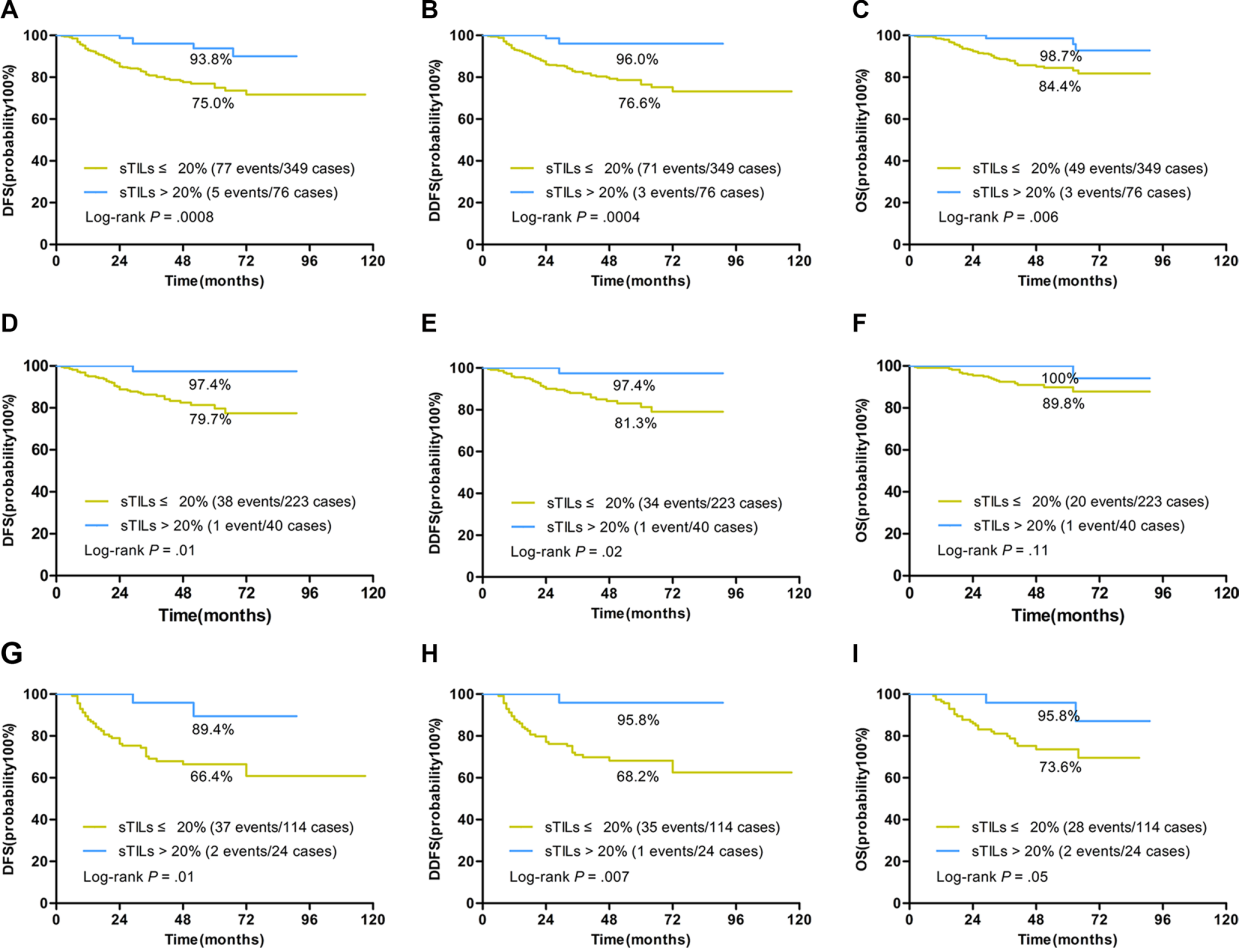
Kaplan-Meier curves for associations of sTILs dichotomized by 20% cutoff with DFS, DDFS, and OS in TNBCs STILs dichotomized by 20% cutoff were significantly associated with prognosis in TNBCs. **(A–C)** Patients with more than 20% sTILs had a 5-year survival rate of 93.8%, 96%, and 98.7% for DFS (A), DDFS (B) and OS (C) in TNBCs. (**D–F)** Patients with more than 20% sTILs had a 5-year survival rate of 97.4%, 97.4%, and 100% for DFS (D), DDFS (E) and OS (F) in node-negative TNBCs. (**G–I)** Patients with more than 20% sTILs had a 5-year survival rate of 89.4%, 95.8%, and 95.8% for DFS (G), DDFS (H) and OS (I) in node-positive TNBCs (Log-rank *P* values were shown).

The average score of sTILs was 14.2% in our study, so all cases were classified as two groups: TNBCs with more than 20% (> 20%) sTILs and TNBCs with no more than 20% (≤ 20%) sTILs. TNBCs with more than 20% sTILs had a significantly better prognosis than TNBCs with no more than 20% sTILs (Figure 4). In univariate analysis, sTILs dichotomized by 20% were significantly associated with DFS (HR 0.24; 95% CI 0.10–0.60, *P* = 0.002), DDFS (HR 0.16; 95% CI 0.05–0.51, *P* = 0.002) and OS (HR 0.23; 95% CI 0.07–0.73, *P* = 0.01) (Table 3). The 5-year survival rate for DFS, DDFS and OS was 93.8%, 96%, and 98.7% respectively in TNBCs with more than 20% sTILs (Figure 4A–4C). In node-negative and node-positive groups, TNBCs with more than 20% sTILs both had a significantly better prognosis than TNBCs with no more than 20% sTILs (Figure 4). The 5-year survival rate for DFS, DDFS and OS was 97.4%, 97.4%, and 100% in node-negative TNBCs with more than 20% sTILs (Figure 4D–4F). The 5-year survival rate for DFS, DDFS and OS was 89.4%, 95.8%, and 95.8% in node-positive TNBCs with more than 20% sTILs (Figure 4G–4I).

**Figure 5:**
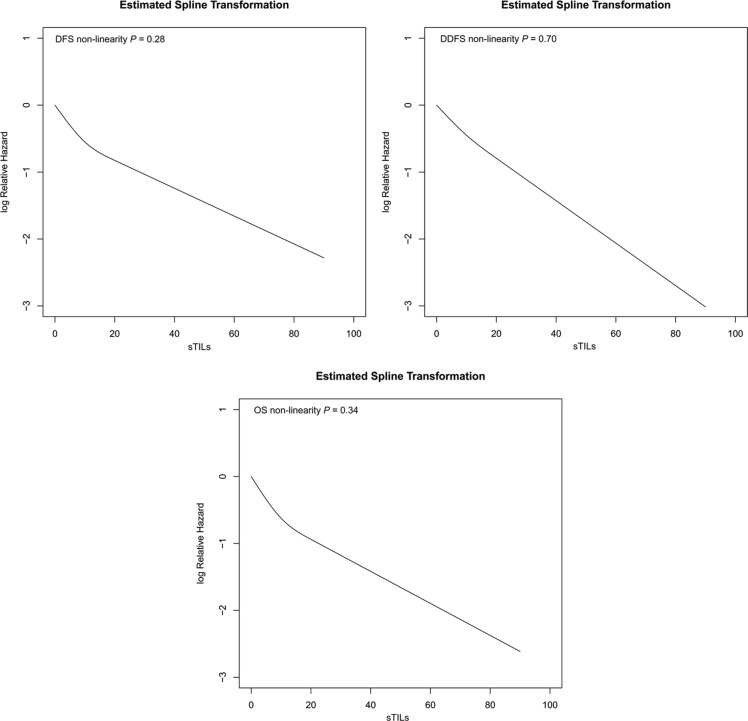
Relationship between continuous sTILs scores and the relative hazard for DFS, DDFS and OS produced by restricted cubic splines models in TNBCs Y axis represented the Log Relative Hazard values; X axis represented continuous sTILs scores. Restricted cubic splines detected no significant departures from linearity between sTILs and relative hazard for DFS (*P* = 0.28), DDFS (*P* = 0.70), and OS (*P* = 0.34).

Multivariate analysis including prognostic variables (age, tumor size, histological grade, and lymph nodes status) confirmed that each 10% increase of sTILs were associated with a 5% reduced risk of first relapse, second primary malignancy or death (HR 0.95; 95% CI 0.93– 0.97, *P* < 0.001), a 5% reduced risk of distant recurrence or death (HR 0.95; 95% CI 0.93–0.97, *P* < 0.001) and a 4% reduced risk of death (HR 0.96; 95% CI 0.93–0.98, *P* = 0.002) (Table 4). Restricted cubic splines models detected no significant departures from linearity between sTILs and hazard ratio for DFS (*P* = 0.28), DDFS (*P* = 0.70), and OS (*P* = 0.34) (Figure 5). Multivariate analysis also confirmed that sTILs dichotomized by 20% cutoff were significantly associated with DFS (HR 0.17; 95% CI 0.05–0.53, *P* = 0.003), DDFS (HR 0.12; 95% CI 0.03–0.49, *P* = 0.003) and OS (HR 0.26; 95% CI 0.08–0.84, *P* = 0.02) (Table 4).

**Table 4:** Multivariate analysis for associations of sTILs with DFS, DDFS and OS in TNBCs

Variables	DFs			DDFs			Os		
	Hr	95% CI	*P*-value	Hr	95% CI	*P*-value	Hr	95% CI	*P*-value
sTILs(per10% increase)	0.95	0.93–0.97	< 0.001^*^	0.95	0.93–0.97	< 0.001^*^	0.96	0.93–0.98	0.002^*^
sTILs (≤ 20% v > 20%)									
sTILs ≤ 20%	1[Reference]		0.003^*^	1[Reference]		0.003^*^	1[Reference]		0.02^*^
sTILs>20%	0.17	0.05–0.53		0.12	0.03–0.49		0.26	0.08–0.84	
Age(years)									
≤ 50	1[Reference]		0.85	1[Reference]		0.76	1[Reference]		0.64
> 50	1.04	0.65–1.70		1.09	0.65–1.80		1.16	0.63–2.12	
Tumor size(cm)									
pT1 (0.1–2.0)	1[Reference]		0.001^*^	1[Reference]		0.001^*^	1[Reference]		0.001^*^
pT2 (2.1–5.0)	1.41	0.85–2.35		1.23	0.72-–2.09		1.41	0.74–2.70	
pT3 (> 5.0)	6.09	2.41–15.39		6.20	2.42–15.89		6.95	2.47–19.520	
Nodal status									
pN0(0)	1[Reference]		0.006^*^	1[Reference]		0.004^*^	1[Reference]		0.02^*^
pN1(1–3)	2.44	1.10–5.40		2.88	1.29–6.42		2.82	1.10–7.28	
pN2/N3(4+)	2.40	1.24–4.66		2.41	1.20–4.85		2.37	1.03–5.47	
Tumor grade									
2	1[Reference]		0.004^*^	1[Reference]		0.002^*^	1[Reference]		0.01^*^
3	2.33	1.30–4.15		2.70	1.43–5.11		2.82	1.30–6.08	

Abbreviations: TNBCs, triple-negative breast cancers; sTILs, stromal tumor-infiltrating lymphocytes; DFS, disease-free survival; DDFS, distant disease-free survival; OS, overall survival; HR, hazard ratio; CI, confidence interval.

* The P value is significant.

## DISCUSSION

Tumor-infiltrating lymphocytes (TILs) have been investigated for a long time as a prognostic factor in breast cancers. It was firstly reported by Aaltomaa et al. in 1992 who found that lymphocytes infiltrates could be a prognostic variable in rapidly proliferating breast cancers [15]. Loi et al. and Adams et al. demonstrated the prognostic role of TILs in TNBCs in 2014 [8–9]. Some studies [5–8, 10] indicated that TILs could reflect the response to chemotherapy and trastuzumab target therapy in TNBCs and HER2-overexpression breast cancers. Although the clinical values of TILs in breast cancers have been recognized gradually, lacking of standardized methodologies for TILs measurement has limited its evaluation and application in practice. Ocana et al. found that there was a significant heterogeneity in TILs evaluation methods in all the identified studies [14]. In 2014, the International TILs Working Group issued consensus recommendations of pathologic assessment methods of TILs, which needed to be validated in multiple laboratories to evaluate its application values. In this study, we performed a retrospective analysis of TILs in 425 TNBCs in a Chinese population with a median follow-up of 4 years, aimed to examine the prognostic role of TILs in TNBCs and to evaluate the feasibility of the scoring methods recommended by International TILs Working Group 2014. Stromal TILs (sTILs) were shown to be an independent prognostic biomarker in TNBCs in our study. Increasing levels of sTILs predicted a significantly lower risk of recurrence or death, distant recurrence, and overall mortality, independent of the known prognostic factors. STILs as a continuous variable by 10% increment were significantly associated with DFS, DDFS and OS. Our study indicated that TILs scored by methods recommended by International TILs Working Group 2014 could be associated with the prognosis of TNBCs.

TILs could be categorized as sTILs and iTILs. ITILs were found in lower scores and detected in 21.6% of the cases, and sTILs were observed in 65.9% of the cases with higher scores in our study. Previous studies also evaluated sTILs and iTILs separately. Loi et al. found that iTILs were associated with prognosis in univariate analysis but not in multivariate analysis, and sTILs were associated with prognosis of TNBCs in both univariate and multivariate analyses [6, 8]. Adams et al. found the association between iTILs and prognosis but did not reach significance, and confirmed that sTILs constituted an independent prognostic biomarker in TNBCs [9]. Ocana et al. analyzed multiple studies reporting iTILs and sTILs. They found a significant heterogeneity in associations of iTILs status with prognosis, and a relatively uniform positive association of sTILs with prognosis [14]. Our study showed that sTILs could be an independent prognostic biomarker in TNBCs, but no association of iTILs with prognosis was demonstrated. Therefore, our study supported the International TILs working group's recommendation of evaluating sTILs as the principal parameter in clinical practice. However, iTILs should still be included in future researches to investigate its potential clinical values.

We also analyzed the LPBC status which was defined as involving ≥ 50% lymphocytic infiltration of either tumor stroma or cell nests suggested by Loi et al. [6]. In our study, only 3.5% of TNBCs were observed to have more than 50% lymphocytes. The associations between LPBC status and DFS, DDFS, and OS were observed but did not reach statistical significance. The term “LPBC” was firstly proposed by Denkert et al, who defined it as tumors with a particularly strong lymphocytic infiltrate [7]. However, the cutoff varied from 50% to 60% among studies, and the associations of LPBC status with prognosis had a significant heterogeneity. Loi et al. and Pruneri et al. found 10.5% and 21.9% LPBC cases respectively and identified a prognostic role of LPBC status for survival [6, 11]. Adams's study found 4.4% LPBC cases and didn't find a significant association between LPBC phenotype with prognosis [9]. The 2014 International TILs Working Group recommendations suggested that it was arbitrary to define 50–60% as the threshold for LPBC [12]. The average score of sTILs was 14.2% in our study, which was similar to Loi's study presented at the 2015 San Antonio Breast Cancer Symposium [16]. In our study, it was shown that sTILs dichotomized by 20% cutoff were significantly associated with the prognosis of TNBCs. TNBCs with more than 20% sTILs had a significantly better prognosis than the patients with no more than 20% sTILs. In view of the limited clinical implication caused by the low proportion of LPBC cases, it was unsuitable to define a cutoff of 50% for the prognostic value of sTILs currently. A cutoff of 20% for sTILs might be more useful in clinical practice. Further research is still needed to obtain an optimal cutoff for TILs in the future.

In our study, sTILs scores were higher in TNBCs with younger age or higher histological grade. Both sTILs and iTILs were found to be positively associated with histological grade. In addition, scores of sTILs were negatively associated with patients' age which hasn't been described in previous studies. The subtle mechanisms of the relationships between TILs and clinicopathologic characteristics remained unknown and the relationships need to be examined in future studies.

The interobserver agreement in TILs evaluation was measured by the intraclass correlation coefficient (ICC) in our study. Scoring of sTILs showed an excellent interobserver agreement, while assessment of iTILs displayed a relatively lower consistency. However, the evaluation methodology was to some extent subjective, and only two pathologists assessed the slides in our study. Therefore, large-scale investigation should be formally performed to assess the intra- and interobserver reproducibility of TILs evaluation before the application of TILs assessment in clinical practice.

Of note, our study was a retrospective analysis based on archived tissues from institutional convenience samples, while some studies [6–10] evaluated TILs in samples from prospective clinical trials. Further large-scale prospective and retrospective studies still need be conducted in independent randomized trials to evaluate the clinical values of TILs in breast cancers.

In conclusion, our study indicated that sTILs scored by methods recommended by International TILs Working Group 2014 were associated with the prognosis of TNBCs. STILs could be an independent prognostic biomarker in TNBCs, increasing sTILs scores predicting better prognosis.

## MATERIALS AND METHODS

### Patients and samples

425 consecutive cases of primary invasive TNBC diagnosed and treated between 2008 and 2012 were extracted from the pathology database of Fudan University Shanghai Cancer Center. The inclusion criteria included: primary invasive TNBCs, available tumor samples, no neoadjuvant therapy before operation, treated with anthracycline or anthracycline + taxanes based adjuvant chemotherapy, available complete clinico-pathological (age, tumor size, grade, lymph nodes status, ER, PgR and HER2 status) and survival data (more than 2-years follow-up). All patients were from a Chinese population. All patients underwent surgery, anthracycline or anthracycline + taxanes based adjuvant chemotherapy, with or without radiation therapy at the Cancer Center. All specimens were fixed with 10% neutral phosphate-buffered formalin and paraffin-embedded. 4 μm-thick slices of representative tumor blocks were stained with hematoxylin and eosin (H&E). Tumors were defined as triple negative as following: < 1% of ER and PgR immunoreactivity, and absence of HER2 protein overexpression or gene amplification. The final median length of follow-up was 4 years (range: 2 years −7.6 years).

### Pathologic assessment

All cases have been reviewed by two experienced breast pathologists (Shui and Yang) to confirm the histological type and grade, according to 2012 World Health Organization (WHO) Classification of Tumours of the Breast [17]. Histopathologic evaluation of TILs was performed by two breast pathologists (Tian, Ruan). The two observers were trained by the evaluation criteria recommended by the International TILs Working Group 2014 [12], and scored each case independently in a blind manner. The mean values of two observers were obtained as final scores for each case.

Histopathologic assessment of percentage of TILs was performed on one representative H&E section of tumor using methods recommended by the International TILs Working Group 2014 [12]. TILs were evaluated within the borders of invasive tumors (including the invasive borders). ITILs were defined as the percentage of lymphocytes within tumor cell nests or in direct contact with the tumor cells (Figure 1A). STILs were defined as the percentage of tumor stroma containing infiltrating lymphocytes (Figure 1A). Areas of *in situ* carcinomas, normal lobules, necrosis, hyalinization and crush artifacts were not included. The results were scored in increments of 10; 0 was defined as < 1%; 10 was defined as 1% to 10%; 20 was defined as 11% to 20% and all other scores were rounded up to the next highest decile. Lymphocyte-predominant breast cancer (LPBC) was categorized as the tumors involving ≥ 50% lymphocytic infiltration in either tumor stroma or cell nests [6].

### Statistical analyses

The associations between TILs (sTILs and iTILs) and clinicopathologic characteristics and prognosis were analyzed. Two types of variables were used to test: one was continuous variables (per 10% increment); the other were binary variables categorized by LPBC-cutoff (50%) and 20% average score cutoff. Differences of TILs as continuous variables between groups were evaluated with Mann-Whitney test and Kruskal-Wallis test. The associations between continuous variables (tumor size, age and nodal status vs TILs) were evaluated with Spearman's rank correlation (*r*). For the survival analyses, the endpoints were disease-free survival (DFS), distant disease-free survival (DDFS) and overall survival (OS). Survival endpoints were defined as the standard definitions proposed by Hudis et al. [18]. DFS was defined as time from date of surgery to date of first relapse (local, regional, contralateral, or metastatic), second primary malignancy, or death resulting from any cause (whichever occurred first). DDFS was defined as time from date of surgery to date of distant recurrence, second primary malignancy, or death resulting from any cause (whichever occurred first). OS was defined as time from date of surgery to date of death (from any cause). Patients who were alive and disease free were censored at date of last contact. Univariate analysis and multivariate COX proportional hazards model were carried to examine the associations between TILs (as continuous and binary variables respectively) and DFS, DDFS and OS. Multivariate COX models were obtained by backward elimination (using likelihood ratio test) in a model containing the main prognostic factors (age (≤50 versus >50 years), tumor size [pT1 (0.1–2.0cm), pT2 (2.1–5.0cm) versus pT3 (>5.0cm)], histological grade (2 versus 3), nodal status [pN0 (0), pN1 (1–3) versus pN2/3 (4+)]. For visualization purposes, Kaplan-Meier estimates were used to produce DFS, DDFS and OS curves. The log-rank test was used to assess differences between groups.

Restricted cubic splines (RCS) models were used to detect the non-linear relationship between TILs (treated as a continuous variable) and the hazard ratio of considered events. In short, the use of restricted cubic splines allows investigation of non-linear effects of continuous covariates in COX model [19–20]. Wald test was used to detect if the complicated RCS model which assumed a non-linear relationship increased the likelihood function when compared to COX model. The *P*-value of the non-linearity test (Wald test) was reported (if *P*-value > 0.05, the null hypothesis of linearity would not be rejected).

The REMARK (Reporting Recommendations for Tumor Marker Prognostic Studies) criteria were followed in this study [21]. A prospective power calculation was based on results by Adams et al. [9] and the sample size of 400 would have a more than 80% power. The interobserver agreement in TILs evaluation was measured by the intraclass correlation coefficient (ICC) using two-way random models. A two-sided *P*-value <0.05 was considered significant. All statistical analyses were performed using the SPSS version 20.0 (Chicago, IL) and R software version 3.2.3 (www.R-project.org).
